# Investigation of Radiochromic Film Use for Source Position Verification through a LINAC On-Board Imager (OBI)

**DOI:** 10.3390/medicina59030628

**Published:** 2023-03-21

**Authors:** Songül Çavdar Karaçam, Duygu Tunçman, Ghada ALMisned, Antoaneta Ene, Huseyin Ozan Tekin

**Affiliations:** 1 Department of Radiation Oncology, Cerrahpaşa Medical Faculty, Istanbul University-Cerrahpaşa, Istanbul 34303, Türkiye; 2 Department of Radiotherapy, Vocational School of Health Services, Istanbul University-Cerrahpaşa, Istanbul 34265, Türkiye; 3 Department of Physics, College of Science, Princess Nourah Bint Abdulrahman University, P.O. Box 84428, Riyadh 11671, Saudi Arabia; 4 INPOLDE Research Center, Department of Chemistry, Physics and Environment, Faculty of Sciences and Environment, Dunarea de Jos University of Galati, 47 Domneasca Street, 800008 Galati, Romania; 5 Medical Diagnostic Imaging Department, College of Health Sciences, University of Sharjah, Sharjah 27272, United Arab Emirates; 6 Faculty of Engineering and Natural Sciences, Computer Engineering Department, Istinye University, Istanbul 34396, Türkiye

**Keywords:** film dosimetry, brachytherapy, quality assurance, LINAC on-board imager

## Abstract

*Background and Objectives:* Quality assurance is an integral part of brachytherapy. Traditionally, radiographic films have been used for source position verification, however, in many clinics, computerized tomography simulators have replaced conventional simulators, and computerized radiography systems have replaced radiographic film processing units. With these advances, the problem of controlling source position verification without traditional radiographic films and conventional simulators has appeared. *Materials and Methods:* In this study, we investigated an alternative method for source position verification for brachytherapy applications. Source positions were evaluated using Gafchromic™ RTQA2 and EBT3 film and visually compared to exposed RTQA radiochromic film when using a Nucletron Oldelft Simulix HP conventional simulator and a Gammamed 12-i brachytherapy device for performance evaluation. Gafchromic film autoradiography was performed with a linear accelerator (LINAC) on-board imager (OBI). Radiochromic films are very suitable for evaluation by visual inspection with a LINAC OBI. *Results:* The results showed that this type of low-cost, easy-to-find material can be used for verification purposes under clinical conditions. *Conclusions:* It can be concluded that source-position quality assurance may be performed through a LINAC OBI device.

## 1. Introduction

There is a tremendous national and international burden caused by cancer, which is a complicated illness with many different causes. Future projections indicate an increase in its prevalence. Researchers predict that by 2040, the annual number of new instances of cancer would rise to 29.5 million worldwide, with 16.4 million deaths attributable to the disease. Research into cancer and its treatment is vital to lessen the dreadful impact of a rising incidence of cancer. Finding better ways to diagnose and detect cancer is another reason that cancer research is important. Tumor biopsies are routinely performed to acquire tissue and cytology specimens for investigation to establish the presence or absence of malignancy. In-depth examination of tumor markers in cancer research has become possible because of the developments in molecular medicine. Cancer detection and characterization have been greatly improved by cutting-edge methods such as ultrastructure, light microscopy, enzyme histochemistry, immunohistochemistry, and molecular diagnostics. These technological advancements have been employed by the cancer research community to better understand the disease and its development. The causes and predisposing for various malignancies may be determined with the use of research studies. On the other hand, cancer research and the development of more effective cancer treatment approaches for the benefit of patients nowadays are two of the most significant pillars of the medical literature. The primary reason for this is the significance of cancer therapy and its direct impact on the patient average lifespan. Treatments based on ionizing radiation in cancer treatment have been used since the beginning of the 19th century, and various techniques have been developed. Brachytherapy is a short-term radiation therapy used to treat malignant/benign tumors by placing radioactive sources on the body surface, in cavities (intracavitary), and/or in tissues (interstitial) [1]. As a result, it has the benefit of protecting the organs at risk (OAR) surrounding the tumor while also providing high-dose therapy to the tumor. During the therapy, radionuclides like Cobalt-60 (^60^Co) or Iridium-192 (^192^Ir) are used as a radioactive source [2]. Brachytherapy methods [3,4] can be categorized according to the amount of radiation exposed, how close the radiation source is to the tumor, and the dose rate. There are five different brachytherapy techniques based on dose rate: Low dose rate (LDR) (0.4–2.0 Gy/h), pulsed-dose-rate (PDR, 0.5–1.0 Gy/h), medium-rate (MDR, 2–12 Gy/h), high-dose-rate (HDR, >12 Gy/h), and ultraLDR (permanent implants, 0.01–0.3 Gy/h) [5]. Particularly for HDR brachytherapy, suitable applicators are positioned in or close to the tumor, which is briefly subjected to HDR radiation. A remote afterloading system applies radioactive sources to the applicators [6]. A remote afterloading machine with an ^192^Ir source is typically used in many radiotherapy centers for brachytherapy treatments. Quality assurance, on the other hand, is essential in all HDR brachytherapy processes [7,8]. The precision of a number of different physical factors including the source activity, implant reconstruction, total dwell duration, and dose calculation method, is critical to the success of brachytherapy treatment since it determines how effective the treatment is. Positioning of the source is another important physical parameter that plays a role in determining the precision of the dose that is administered to the patient [7,8,9]. It is essential to control the source dwell position compatibility of the dummy source and the source positions before adopting a new source. There have been several reports of different techniques that may be used to test the positional accuracy of HDR remote afterloading devices [10] such as the autoradiograph, the double exposure radiograph, and the closed-circuit TV camera techniques. In recent years, a number of various techniques for source control have been used, and their usage has been determined by the device manufacturer. For the purpose of halting position control devices, some clinics make their own phantoms, which come in a variety of forms. Autoradiography is a classic example of a universal approach that has been used for many years in LDR as well as HDR settings. The radiographic localization of sources with this method involves making exposures on a single film, with two exposures taken with the X-ray tube and afterloading machine. Visualization is essential with this technique. On the film, a dark dot indicates the source, which should fall on the line between two white dots. X-rays and film are needed for the autoradiograph method [11]. Conventional simulators used such as X-ray sources and radiographic films have been replaced by computed tomography simulators and digital film technology in our clinic. The Gammamed 12i 3/24 HDR afterloader does not have a camera system. Most of the literature describes the evaluation of source position control through a conventional simulator by exposing the radiographic film. In the literature, however, there are no explanations on how to do such control with external devices in radiotherapy departments and solve the source position control. Accordingly, we aimed to evaluate radiochromic film for source positioning quality control when irradiated with a Varian Medical Systems on-board imager (OBI) instead of a conventional radiotherapy simulator. The finding of the current investigation may be useful for cost-effective, and rapid solutions toward obtaining higher quality levels for source positioning purposes in radiotherapy departments.

## 2. Materials and Methods

**Figure 1:**
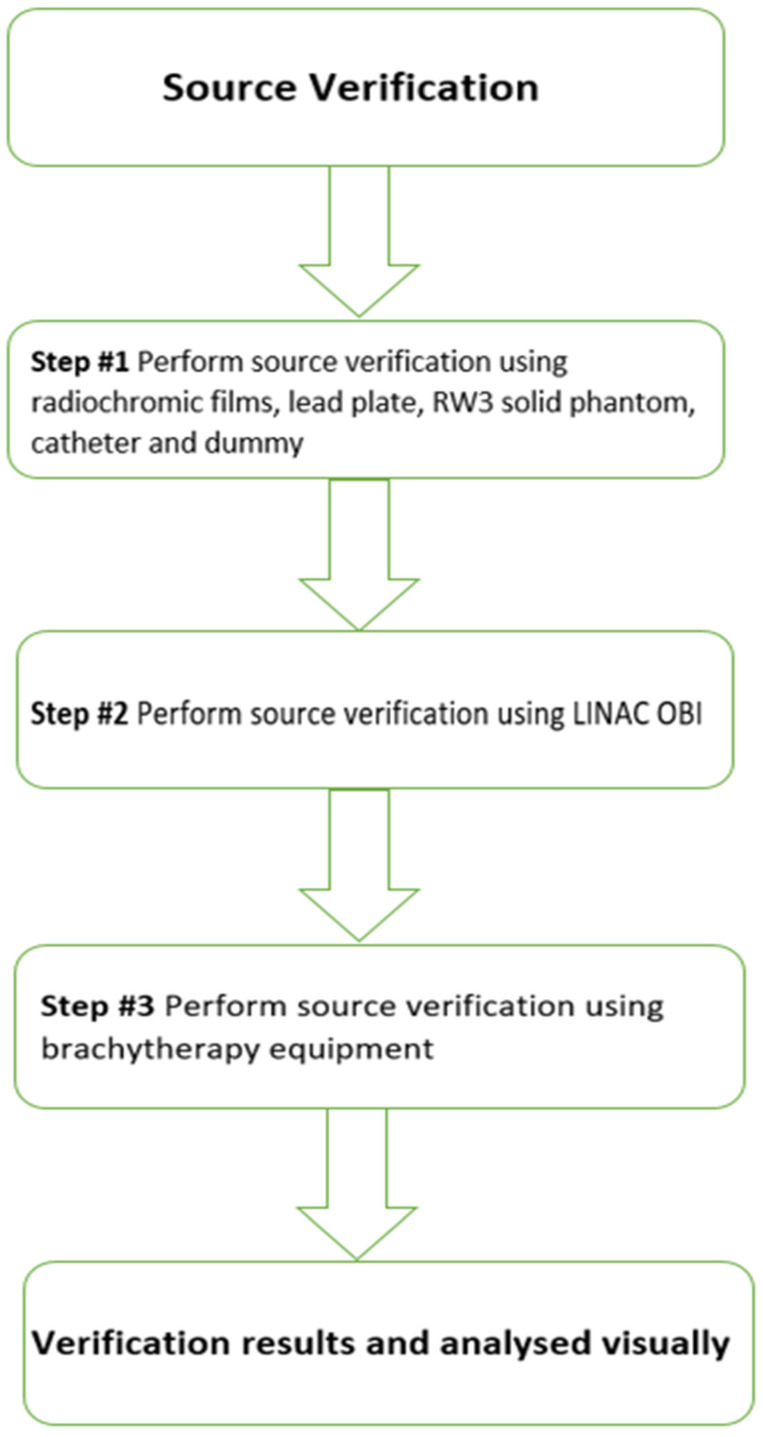
Study design.

Figure 1 illustrates the study design process.

**Figure 2:**
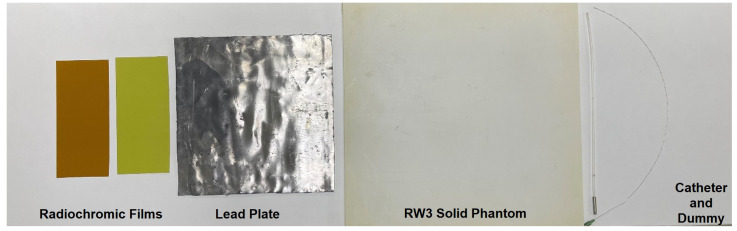
Source verification equipment.

Verification of the source position requires Gafchromic™ EBT 3 film for brachytherapy, Gafchromic RTQA2 film for LINAC, a lead plate, RW3 solid phantom, a catheter, and a dummy source (Figure 2).

**Figure 3:**
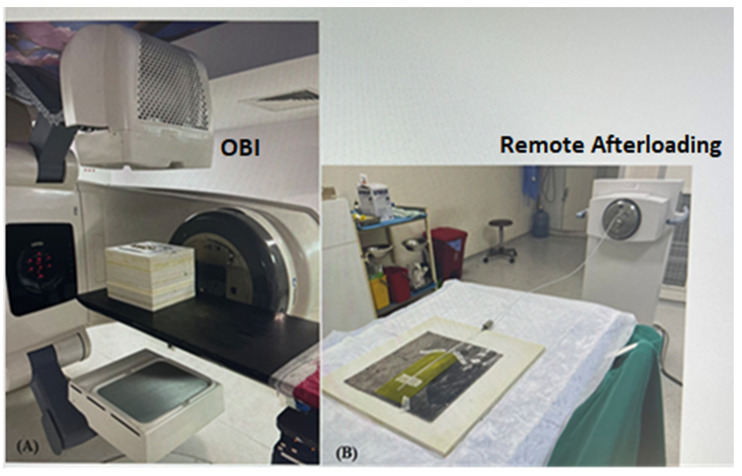
(**A**,**B**). Configuration of OBI and remote afterloading.

Varian’s kV imaging device (Varian Medical Systems, Inc., Palo Alto, CA, USA) is referred to as the OBI system. This system allows clinicians to image and treat patients using image-guided radiotherapy (IGRT) with a LINAC that isocentrically rotates around the patient. The system includes a flat panel detector (KVD) and a kV X-ray source (kVS) that can provide images in pulsed fluoro and radiographic modes using x-rays. We used the radiographic mode. Other irradiations were performed on a remote-controlled mobile Gammamed 12-i plus 3/24 model (Varian Medical Systems, Charlottesville, VA, USA) HDR brachytherapy unit. This equipment has one stepping source with an active length of 3.5 mm and an active diameter of 0.6 mm. The radiochromic films used in this study were Gafchromic RT QA2-1010 (Lot #: 11171701) and Gafchromic EBT3 (Lot #: 05112103). Gafchromic RTQA2 film is most commonly used for alignment of the radiation light/field test for the LINAC. Gafchromic EBT3 film (Ashland Specialty Materials, Bridgewater, NJ, USA) is generally used for plan verification in external radiotherapy, but is also used in brachytherapy applications [12]. The measurements were performed in two steps. In the first step, a 2-mm thick lead sheet was prepared and placed under the Gafchromic film to provide more backscatter. The catheter was placed on the film. The assembly was placed on the LINAC couch, and the film was centered on the radiation field. An X-ray exposure was made with the Varian Clinac OBI with a dummy source marker inserted (Figure 3A). A tungsten dummy source catheter with 1 cm spacing was used. Gafchromic films were exposed with different kVp, mA, and exposure time combination parameters to define the optimal film exposure quality. The best combination was 80 kV and 250 mA for radiography. However, different exposure times (s) were defined because the Gafchromic films had different properties. The second step was to connect the catheter to the HDR device while keeping it fixed to the film (Figure 3B). To define the best combination in terms of contrast and brightness, the Gafchromic films were exposed with different dwell times. The best combination was a dwell time of 0.5 s for RTQA2 film, 0.8 s for EBT3 film, and a step size of 1.0 cm for autoradiography.

**Figure 4:**
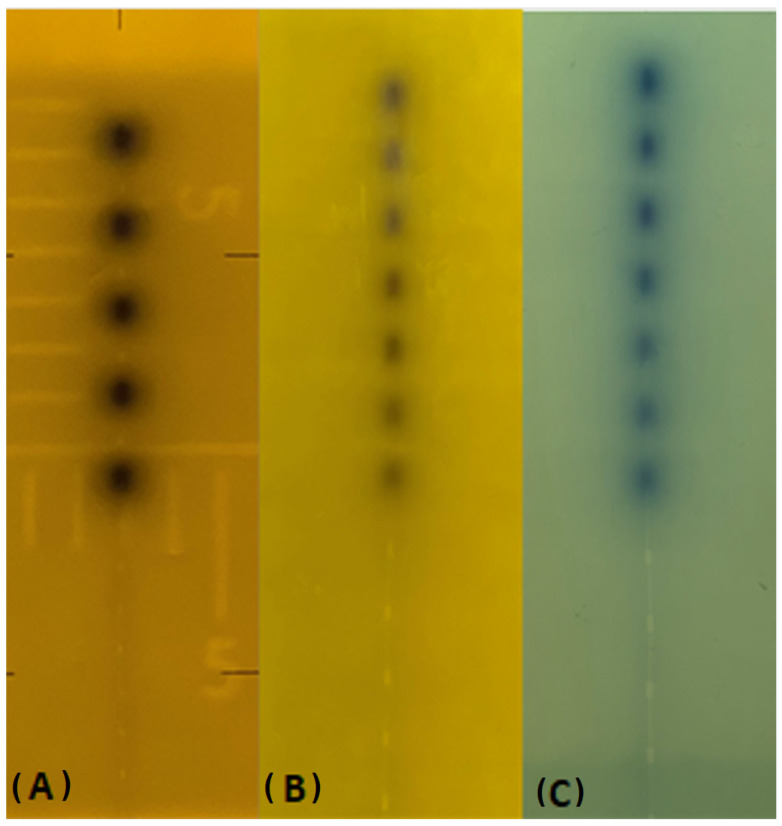
(**A**–**C**). Appearances of the irradiated films. The first film (**A**) was irradiated in a conventional simulator. The second and third films (**B**,**C**) were irradiated on the LINAC OBI.

As seen in Figure 3A, the catheter was placed on Gafchromic RTQA2 film and a lead sheet onto a slab of the solid water phantom. Additional slabs of solid water phantom were placed onto a LINAC treatment couch. The kV tube was positioned at a 00-gantry position, and the Gafchromic film was irradiated. Moreover, the catheter was placed on Gafchromic film and a lead sheet onto a slab of solid water phantom, was moved to the brachytherapy room and connected to the brachytherapy device, where the Gafchromic film was irradiated (see Figure 3B). All investigations in this study were performed with GammaMed Plus 3/24 flexible implant tubes (6 F, 50 cm, blind end). Figure 4 shows the autoradiograph of the Gammamed 12-i ^192^Ir source on two types of film. A comparison was made between the irradiated radiochromic film using a conventional simulator. Visual inspections were used for both film types. We expected the distance between the center of the source and tungsten in <±2 mm.

## 3. Results and Discussion

Radionuclides and improvements in remote afterloading devices have stimulated renewed interest in brachytherapy. The verification of HDR source-positioning accuracy (or the indexer length) and of security equipment is a fundamental part of good HDR quality assurance [1,4]. Independent checks are standard practice in external beam radiotherapy. For brachytherapy, an independent check is also desirable, but there is no generally accepted method for doing so. Meanwhile, each method uses various detectors or systems. Currently, there is no technique in clinical usage that can offer the level of quality control that is sought of the source. Kilovoltage radiography, also known as the conventional simulator, is used in the conventional method to image the dummy source markers, which is then followed by autoradiography with active sources on a single film using the same geometry for the applicator. This method is used to verify the accuracy of the source position. This study provides a novel methodology in which the dummy source markers are scanned using an external irradiation device known as an OBI, and their dwell locations are described with respect to an applicator-defined axis. The autoradiograph of the source was carefully placed on the sites of the dummy source at intervals of one centimeter. For each film, the source coordinates were determined by tracing black dots over the image. The RTQA2 film has been typically developed for use in LINAC device quality assurance, in addition to location verification for HDR and autoradiography of implanted seeds, plaques, and other sources. Due to the fact that EBT3 film is often used in a clinical context for dosimetric objectives, we additionally researched whether or not it could be utilized for the identical objective. In terms of image, both Gafchromic films were determined to be comparable in a visual assessment; however, in terms of irradiation timings, EBT 3 films required five times longer irradiation than standard Gafchromic films (see Table 1). Table 1 and Table 2 describe each conceivable irradiation condition for every single film type. In a similar manner, we extended the dwell duration in the brachytherapy device (see Table 2). Figure 4 displays images from each of the three types of film that were analyzed for this investigation. There were no obvious variations found between the films that had been taken and stored in the past using the traditional simulator and the films that had been irradiated utilizing the OBI device. The report of the AAPM (American Association of Physicists in Medicine) Task Group 56 published recommendations for the positional and temporal accuracy of remote afterloading equipment and dose delivery accuracy for HDR source brachytherapy [11].

They mentioned that verification of the source position requires that one confirms that the intended sequence of active sources or dwell positions is delivered to the correct position for the applicator. Application quality control tests are difficult during treatment. Hence, it is necessary to implement quality control tests before treatment. There is no widely accepted method for verifying the source position in HDR brachytherapy. It is known that HDR brachytherapy devices or clinic-specific methods such as a charge-coupled device (CCD) cameras and multi-slit phantoms [12,13,14,15,16] are used for source position control. However, these systems are only available in some clinics.

Radiation oncology clinics use a LINAC device for external therapy and brachytherapy for internal therapy; most clinics have both types of these devices. For this reason, we aimed to develop an additional method that could be used in all radiotherapy clinics. In our study, exposed films by a Varian OBI were compared with conventional simulators using two different types of radiochromic film, RTQA2 and EBT3. Radiochromic films are an effective tool for HDR brachytherapy quality assurance. The films are designed to change color when a specified radiation dose is approached and can be cut to the size of the catheter with a margin. It is also easy to transport radiochromic films. Many authors have also recommended the use of conventional simulators for source visualization [5,7]. Various researchers have conducted practical studies using radiographic films to show the source position accuracy of different techniques. Evans et al. [9] demonstrated the use of three film options (XV-2radiographic film, EBT radiochromic film, XR-QA radiochromic film) in a brachytherapy auto-radiograph quality assurance application and evaluated the performance of films exposed by a conventional simulator. Stern et al. [16] investigated the actual source dwell positions for two sets of ring applicators with a GammaMed HDR afterloading device. KODAK EDR2 Ready-Pack radiographic film was exposed using a conventional simulator autoradiographic method. In previous years, conventional simulators were used for the autoradiographic method; however, they have been replaced by computed tomography simulators. Radiographic film has also been replaced by digital film technology. In addition, due to the high sensitivity of radiochromic films in the low energy range, the HDR brachytherapy source position makes it an ideal option for quality assurance.

## 4. Conclusions

Trying to cure cancer and returning the patient to a healthy life span is the ultimate goal of cancer therapy. This might be acceptable or unattainable depending on the patient’s condition. The basic purpose of cancer therapy is to eliminate the disease permanently. The term primary therapy refers to the first phase of therapy for cancer, and for the most prevalent cancers, surgery is the most often utilized primary treatment. Radiation therapy or chemotherapy may be used as the main treatment if the cancer is highly susceptible to any of these methods. Adjuvant therapy is given after initial treatment in an effort to eliminate any lingering cancer cells and prevent a relapse. An adjuvant therapy may be added to any cancer treatment. Chemotherapy, radiation treatment, and hormone therapy are all examples of common adjuvant therapies. Neoadjuvant therapy is quite similar, but it involves therapies that are administered before the main treatment in order to facilitate or improve the primary treatment. Therapeutic measures aim at alleviating suffering. Palliative care is aimed at reducing discomfort for patients, whether that is from the disease itself or through therapy for it. Symptoms may be treated with a variety of surgical procedures, radiation treatment, chemotherapy, and hormone therapy. Pain and shortness of breath are only two examples of symptoms that may be treated with different drugs. Cancer patients may employ palliative care in addition to curative therapy. The overall success of cancer therapy is closely related to numerous technical details of the process. These technical differences not only affect the level of the response at the conclusion of the therapy, but shaping this response in a positive direction also has a direct impact on the patient’s quality of life. Among the technical details of the treatment process, the timing and delivery of the source to the required dwell location must be carried out with the utmost precision due to the high radiation rate connected with HDR treatments. The administration of dose to the proper treatment volume or an undesired dose distribution will result from incorrect dwell location positioning. A variety of factors including an inappropriate catheter length, an inadequate transfer tube length, a fluctuating source position, a malfunction of remote afterloading system, and an inaccurate treatment setup and planning, can lead to incorrect source placement [17]. The most common isotope is ^192^Ir, which has a half-life of 74 days; hospitals, therefore, renew their sources at intervals of around three months. The positional accuracy of a new source should be verified by testing the ability of the unit to drive the source to a desired position [10]. The method described in this study was developed to provide a positional verification of an ^192^Ir source by adapting the autoradiographic method to technological developments. In this study, we proposed an alternative method to evaluate the mechanical accuracy of an HDR source position using Varian’s on-board imager device with radiochromic films. It was shown that mechanical control of the source position using radiochromic film exposed to an OBI device is usable and reliable. In order to conduct a visual investigation of the source location, the images provide clear indications of where the source is located. In any case, it allows for data to be collected using this method in all brachytherapy units as well as for any and all radiation units that have OBI. Since it does not need any processing after being exposed to radiation, radiochromic film may be used as a substitute to conventional radiographic film, or at least can be used as a backup method. In addition, in comparison to traditional radiographic film, which is light-sensitive and needs chemical development processes, this film offers a substantial number of advantages.

## Figures and Tables

**Table 1:** Parameters for OBI.

Film	SSD (cm)	kV	mA	ms
RTQA2 film	70	80	250	1000
EBT 3 film	70	80	250	5000

SSD = source skin distance, cm = centimeter, kV = kilo voltage, mA = milli ampere, ms = milli second.

**Table 2:** Parameters for HDR remote afterloading unit.

Film	Dwell Time (s)	Dwell Position Interval (cm)	Total Dwell Time (s)
RTQA2 film	0.5	1.0	3.5
EBT 3 film	0.8	1.0	5.6

s = second, cm = centimeter.

## Data Availability

Data available upon reasonable request.
